# A Lightweight AlTiVNb High-Entropy Alloy Film with High Strength-Ductility Synergy and Corrosion Resistance

**DOI:** 10.3390/ma15238568

**Published:** 2022-12-01

**Authors:** Xiaobin Feng, Chuangshi Feng, Yang Lu

**Affiliations:** 1 Hubei Key Laboratory of Theory and Application of Advanced Materials Mechanics, Wuhan University of Technology, Wuhan 430070, China; 2 Songshan Lake Materials Laboratory, Dongguan 523808, China; 3 Department of Mechanical Engineering, City University of Hong Kong, Hong Kong 999077, China; 4 Nano-Manufacturing Laboratory (NML), Shenzhen Research Institute, City University of Hong Kong, Shenzhen 518060, China

**Keywords:** AlTiVNb, lightweight high-entropy alloy film, amorphous structure, strength-ductility synergy, corrosion resistance

## Abstract

The simultaneous improvement of mechanical and corrosion resistance is of great significance for engineering applications. In this work, a novel lightweight amorphous structure AlTiVNb high-entropy alloy (HEA) film was fabricated by magnetron sputtering. The compression test of the AlTiVNb HEA film nanopillar exhibits a high compressive strength of up to 3.6 GPa and deformability approaching 58%. The high strength is affected by the disordered state, the nanostructure, and the lattice distortion effect, while the high ductility comes from the ductile shear band and the island structure. In addition, the AlTiVNb HEA film shows a current density of 4.90 × 10^−8^ A/cm^2^ and a potential of −0.234 V in the 3.5% NaCl solution, comparable to that of the 316L stainless steel. The chemical disorder state, cocktail effect, and homogeneous amorphous structure contribute to excellent corrosion resistance. This finding offers new insights into high-performance HEA films with robust mechanical and anticorrosion performances for microelectronic devices and mechanical metamaterials.

## 1. Introduction

Since the concept of high-entropy alloys (HEA) was proposed in 2004, HEA has become a research focus in the materials science and engineering fields [1,2,3,4]. HEAs inherit four main characteristics, namely the high-entropy effect, slow diffusion effect, lattice distortion effect, and cocktail effect [5], manifesting huge potential research and engineering applications. At present, the pursuit of preparing lightweight alloys has become an important research direction to meet the demand of practical engineering applications. Some low-density elements can be considered to fabricate lightweight HEA, such as Al, Be, and Li, etc [6]. Among these candidates, Huang et al. prepared lightweight AlTiVCr-based HEAs via microalloying, which presented a high hardness and low density [7]. Stepanov et al. studied the structure and mechanical properties of the lightweight single BCC phase structure AlNbTiV HEA with a density of 5.59 g cm^−3^ [8], showing a high hardness of 4315–4394 MPa and excellent compressive yield strength of 1 GPa and 685 MPa at room temperatures and 800 °C, respectively. Despite the fact that these lightweight HEAs are not lighter than some alkali and alkaline earth metals, lightweight HEAs provide more balanced mechanical properties and low manufacturing costs [7,9,10,11].

The HEA films usually present more features of simple preparation, easy adjustment of composition, cost saving, and rapid high-throughput screening than the corresponding bulks. Chen et al. first prepared the nanostructured multi-element HEA nitride films by the reactive direct current (DC) sputtering method, which presents a single-phase structure and excellent electrical and mechanical properties [12]. Within the pioneering research, HEA films present excellent mechanical properties, electrical properties, corrosion resistance, and high-temperature properties [13,14,15]. For example, Tsai et al. prepared amorphous and BCC phase structure (AlCrMoTaTi)N coatings via magnetron sputtering. The hardness and modulus reached maximum values of 30.6 GPa and 291.6 GPa, respectively, by increasing the N_2_-to-total (N_2_+Ar) flow ratio to 30% [16]. Feng et al. found that the Al_0.1_CoCrFeNi film manifests a high strength over 4.0 GPa and a compressive strain exceeding 15%, which were mainly affected by the high-density stacking and nanotwins [17]. Zheng et al. studied the passivation behavior of a novel VAlTiCrSi amorphous film in artificial sea water. The corrosion resistance of the amorphous film is much higher than that of the 304 stainless steel, which is attributed to the complex composition [18]. In view of the outstanding performance, HEA films are expected to apply in hard coatings, corrosion-resistant coatings, diffusion barriers, and microelectromechanical systems (MEMS). Especially by decreasing the thickness of HEA films on micro-/nanolattices, the fracture of the strut can be delayed, leading to a higher specific strength [19,20].

In contrast, many studies have demonstrated that the HEA films tend to be a single-phase solid solution crystal or an amorphous structure due to the high-entropy effect and slow diffusion effect [21,22,23,24]. Among these structures, the amorphous HEA films tend to show higher corrosion resistance. Zheng et al. prepared VAlTiCrSi amorphous film and found that the low corrosion current density of 4.68 × 10^−9^ A/cm^2^ is affected by the amorphous microstructure and anti-corrosion constituent elements [21]. Generally, the formation of the amorphous is affected by the elemental composition and high cooling rate. Braeckman et al. found that the phase structures of the Nb_x_-CoCrCuFeNi HEA films change from crystalline to amorphous with the increase of Nb content [22]. Feng et al. prepared the amorphous structure WNbMoTaV film, which possesses a low glass-forming ability through pulsed laser deposition [23]. This phenomenon is mainly affected by the super high cooling rate under the deposition. In terms of mechanical properties, it is generally believed that HEA films with the amorphous structure possess high hardness. Muftah et al. produced a quaternary amorphous FeCrSiNb HEA film with a hardness of 15 GPa and a reduced Young’s modulus of 229 GPa, which is due to the uniform and single-phase structure and the addition of Nb elements [24].

However, the strong HEA films mentioned above suffer from low ductility in general. How to balance the strength and ductility of amorphous HEA films is important for their applications in the field of flexible microelectronic devices and mechanical metamaterials. In addition, seeking a high-performance HEA film with low density and corrosion resistance is of great significance for their applications in extreme conditions. In these cases, it is crucial to develop a high hardness, high strength–ductility synergy, and excellent corrosion resistance of amorphous lightweight high entropy alloy film. In this work, the lightweight AlTiVNb HEA film was prepared by magnetron sputtering, and the microstructure, mechanical properties, and corrosion resistance were investigated.

## 2. Experimental Methods

The AlTiVNb HEA thin films were performed using the high-vacuum magnetron sputtering system (JCP500, Beijing Technol Science, Beijing, China) onto the (100) oriented single-crystal Si wafer substrates (2 inch in diameter). The target was fabricated by casting with a nominal composition of AlTiVNb (1:1:1:1, at.%) with a purity higher than 99.9%. Before deposition, the Si wafer and target were cleaned with deionized water, acetone, and ethanol for 10 min, respectively, then dried with clean nitrogen. The directed current power of 100 W was set as the sputtering energy, and the flow of the sputtering atmosphere (Argon, purity ≥ 99.999%) was chosen as 30 sccm. The work distance and work pressure were set as 90 mm and 0.5 Pa, separately.

The surface and cross-sectional microstructures of the deposited film were observed by scanning electron microscopy (SEM; Supra 55 Sapphire, Carl ZEISS, BW, Germany) and the attached energy dispersive X-ray spectrometer (EDS) was used to detect the chemical composition. The three-dimensional morphology and surface roughness were acquired by atomic force microscope (AFM, Dimension Icon, Bruker, BW, USA) and the crystal structure was characterized by X-ray diffraction (XRD; Smartlab, Rigaku, Tokyo, Japan) with the film mode. The inner structure was observed by Transmission Electron Microscopy (TEM; JEM 2100F, JEOL, Japan) equipped with Selected Area Electron Diffraction (SAED). Nano-indentation (TI950, Hysitron, Billerica, MA, America) with a triangular pyramid Berkovich indenter following the Oliver–Pharr method is used to detect the hardness and modulus [25]. The loading–holding–unloading time was set as 5 s, 2 s, and 5 s, respectively, and the experiment was conducted nine times to obtain the average hardness and modulus. The HEA film pillars with a height–diameter ratio of 2–3 were prepared by Focused Ion Beam (FIB; Scios DualBeam, FEI, Columbia, MD, USA). The nano-indentation tests were performed on HEA film pillars with a flat indenter and a loading rate of 5 nm/s, and the experiment was repeated at least three times to obtain more accurate data. Tafel curve testing was conducted using a standard three-electrode system with a Pt counter electrode and a saturated Ag/AgCl reference electrode via an electrode electrochemical workstation (CHI760E, Chen Hua, China). The 3.5 wt.% NaCl solution was used as the corrosion medium at room temperature and the scanning rate was set as 1 mV/s from an initial potential of −0.5 V to 1.5 V. The corrosion area was cut as 1 cm^2^ for easy calculation.

## 3. Results and Discussion

**Figure 1:**
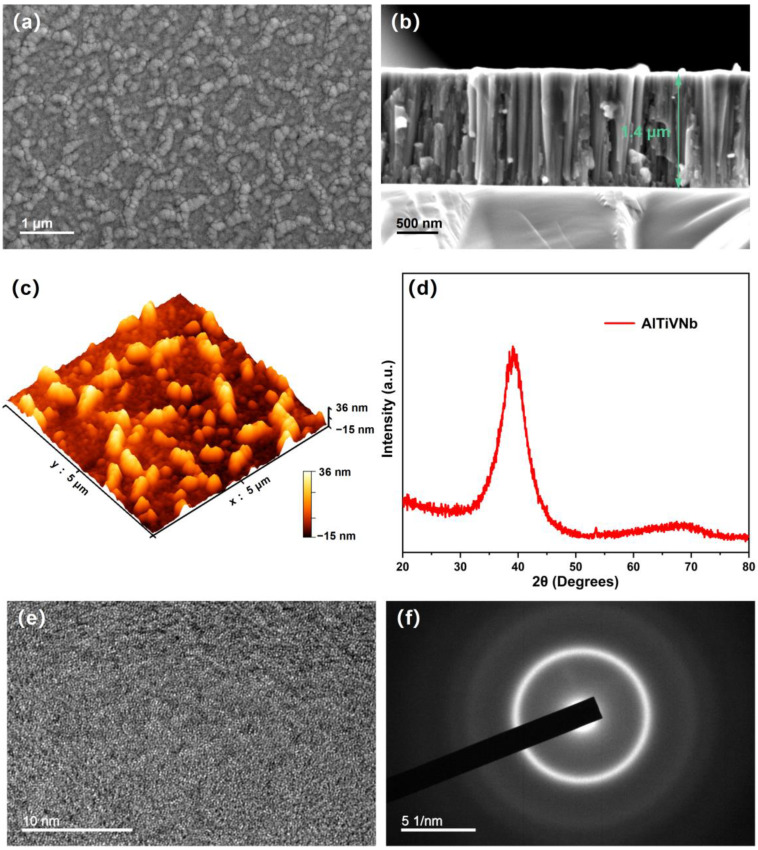
The morphology and phase structure of the AlTiVNb HEA film. (**a**) The SEM surface morphology of the film; (**b**) Cross-section morphology; (**c**) 3D surface topography by AFM; (**d**) XRD pattern, showing an amorphous structure; (**e**) The bright field HRTEM image of the film; (**f**) The corresponding SAED patterns.

To investigate the morphology and phase of the AlTiVNb HEA film, the microscope observations (both SEM and AFM) and XRD detection were performed on the AlTiVNb HEA film. Figure 1a,b show the surface and cross-sectional morphologies of the AlTiVNb HEA film. It shows that a number of island structures with approximately several hundred nanometers width formed on the surface irregularly and formed a clear boundary between each island. The film thickness is measured to be 1.4 μm. The cross-sectional observation also shows a good bonding between the HEA film and substrate. The surface fluctuation of the island structure is 7.5 ± 1.8 nm, as shown in Figure 1c, illustrating the smooth surface and the high-quality film deposition by magnetron sputtering. The XRD pattern of the film presented in Figure 1d show typical broad peaks, indicating an amorphous structure. To further study the microstructure of the AlTiVNb HEA film, the bright field high-resolution transmission electron microscopy (HRTEM) image and the SAED pattern are shown in Figure 1e,f. It can be seen that no obvious lattice fringe is observed, and the diffraction ring shows an amorphous structure, which is consistent with the XRD result.

**Figure 2:**
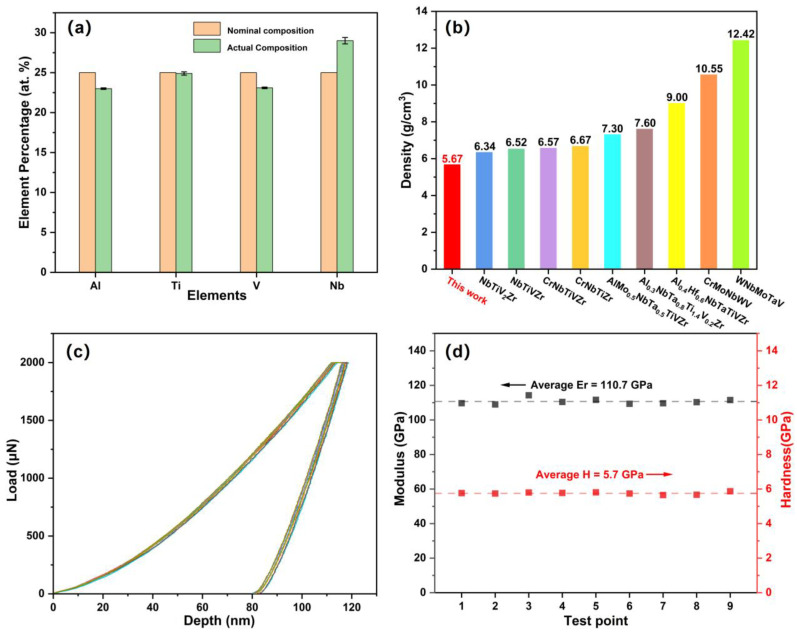
(**a**) The element percentage (at. %) of the AlTiVCr HEA film; (**b**) The density of the AlTiVCr HEA film analogous to HEAs including NbTiV_2_Zr [26], NbTiVZr [26], CrNbTiVZr [26], CrNbTiZr [26], AlMo_0.5_NbTa_0.5_TiVZr [26], Al_0.3_NbTa_0.8_Ti_1.4_V_0.2_Zr_1.3_ [27], Al_0.4_Hf_0.6_NbTaTiVZr [27], CrMoNbWV [28], and WNbMoTaV HEAs [28]; (**c**) The load–depth curves of the AlTiVCr HEA film; (**d**) The corresponding modulus and hardness.

The element contents of the film measured by EDS compared to the nominal composition are shown in Figure 2a. The contents of the four elements are close to the initial design composition. Accordingly, the density of the thin film can be calculated via the rules of mixtures, 5.67 g cm^−3^, which presents the lower density among reported similar HEAs composition, as shown in Figure 2b, including NbTiV_2_Zr [26], NbTiVZr [26], CrNbTiVZr [26], CrNbTiZr [26], AlMo_0.5_NbTa_0.5_TiVZr [27], Al_0.3_NbTa_0.8_Ti_1.4_V_0.2_Zr_1.3_ [27], Al_0.4_Hf_0.6_NbTaTiVZr [27], CrMoNbWV [28], and WNbMoTaV HEAs [28]. To study the mechanical properties of the AlTiVNb HEA film, the load–depth curves derived from the nanoindentation are shown in Figure 2c and the modulus and hardness of the film are presented in Figure 2d. The curves show high repeatability, illustrating that the film is dense and uniform. From Figure 2d, it can be seen that the average modulus is about 110.7 GPa and the average hardness is about 5.7 GPa, which shows a consistent modulus but higher strength compared to the corresponding bulk [8]. Some studies have reported that the hardness of the HEA film is much higher than that of the bulk, which is mainly affected by the nanostructure and density [29,30]. Therefore, the high hardness of the presenting amorphous AlTiVNb HEA film contributes to the single phase, the uniformity of the surface structure, and the chemical disorder state.

**Figure 3:**
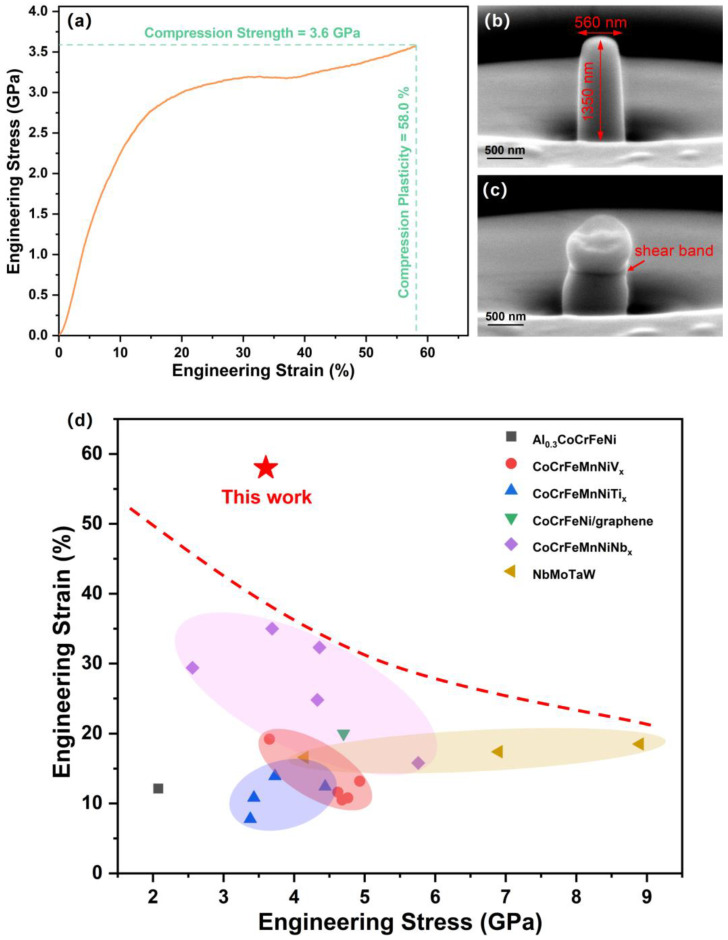
(**a**) The compressive engineering stress–strain curve of the AlTiVCr HEA film; The corresponding morphologies of HEA film pillars before (**b**) and after (**c**) compression; (**d**) The engineering strain and stress of the AlTiVCr HEA film pillar compared with other HEA pillars with similar scales in diameter.

Generally, amorphous structure materials tend to possess a brittle mechanical characteristic due to the chemical disorder and non-grain boundary structure which provide a facile propagation path for crack propagation. However, some interesting phenomena occur in the presenting film. Figure 3 shows the compressive engineering stress–strain curves and the corresponding pillar morphologies before and after deformation. It can be seen from Figure 3a that the film presents a high strength of 3.6 GPa and an excellent plasticity of 58.0%. Before deformation, the pillar was fabricated into a normative size (560 nm in diameter and 1350 nm in height) to ensure the data reliability, as shown in Figure 3b. After deformation, the pillar becomes significantly extruded and forms a distinct shear band in the middle, as shown in Figure 3c. The engineering stress and strain of the AlTiVCr HEA film pillar compared with other related HEA films with similar dimension are shown in Figure 3d, including Al_0.3_CoCrFeNi [31], CoCrFeMnNiV_x_ [32], CoCrFeMnNiTi_x_ [33], CoCrFeNi/graphene [34], CoCrFeMnNiNb_x_ [35], and NbMoTaW HEA pillars [36] which present higher strength and plasticity than other HEA-based pillars.

Combined with the microstructure and the engineering strain–stress curve, one can conclude that the amorphous HEA film presents excellent mechanical properties, especially plasticity. The high strength is due to the disordered state, the nanostructure, and the lattice distortion effect caused by the different atomic sizes. The high plasticity can be summarized below. Different from crystalline materials, amorphous materials do not yield dislocations during deformation, showing a highly localized feature. Therefore, the amount of plastic strain is concentrated in the shear band with a thickness of only tens to hundreds of nanometers [37]. Many studies focus on the effect of the shear band on the deformation of metallic glasses, but the specific mechanism is still unclear [38,39,40]. Generally, there are two types of shear bands in metallic glasses, one is the ductile shear band with accelerated–decelerated–stopped shear band expansion, and the other is the brittle shear band with rapidly accelerated evolution after the shear band expansion starts. The ductile shear bands control the stable plastic flow of the amorphous alloy, while the brittle shear bands control the macroscopic brittle failure behavior of the amorphous alloy [41]. In this work, the HEA pillar presents a large plasticity, and the presenting shear band may belong to the ductile shear band. Otherwise, the island structure may disperse more deformation energy and the evenly-distributed void may hinder the crack from deformation.

**Figure 4:**
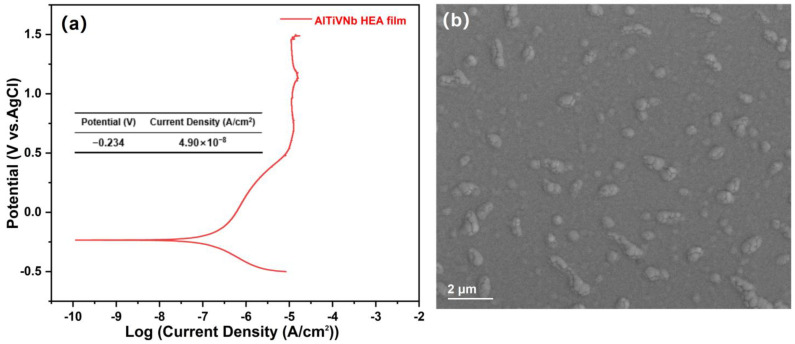
(**a**) The Tafel curves of the film in the 3.5 wt.% NaCl solution; (**b**) The surface microstructure of the film after corrosion.

Corrosion resistance is also an important merit for microelectronic devices and mechanical metamaterials to meet the application requirements of various environments. Figure 4 shows the Tafel curves of the AlTiVNb film and the surface microstructure after corrosion. In Figure 4a, the film shows a current density of 4.90 × 10^−8^ A/cm^2^ and a potential of −0.234 V. Compared with the 316L stainless steel in our previous work, which has a current density of 1.01 × 10^−7^ A/cm^2^ and a potential of −0.263 V [23], the film shows a higher potential and higher current density, which illustrates that the film presents an equivalent corrosion resistance as 316L stainless steel. In addition, the surface microstructure presents relatively complete morphological features with no pitting pits, as shown in Figure 4b.

According to the rule proposed by Inoue, the multicomponent system can promote the glass-forming ability by decreasing the Gibbs free energy [42]. In addition, the high cooling rate and sluggish diffusion effect decrease the atom mobility in the multi-component system, further promoting the formation of an amorphous structure [23]. It is worth mentioning that the island structure appears on the surface of the amorphous film, which is completely different from the amorphous bulk. This characteristic can be explained by the coalescence-induced void formation mechanism (CVF), which was proposed by Lloyd et al. [43] and Nakahara [44], as well as the shadowing effect proposed by Thornton et al. [45,46]. In the initial deposition stage, low-mobility adatoms are agglomerated and the amorphous cluster is nucleated on the substrate. Then, the clusters grow as the island growth mode and form high-density small voids. With the continuous deposition, the amorphous cluster coalesces and grows in the direction perpendicular to the substrate, and large voids form during this columnar structure growth. The small-scale, high-density voids affect the surface and cross-sectional morphology, and the island structure formed. It is worth mentioning that part of the island structure disappeared after corrosion, which implies that the island structure induces a more specific surface area in the 3.5 wt.% NaCl solution. The excellent corrosion resistance may be ascribed to the chemical disorder state, cocktail effect, and homogeneous amorphous structure.

## 4. Conclusions

In summary, the amorphous AlTiVNb HEA film with a thickness of 1.4 μm was delicately prepared by magnetron sputtering. The density of the HEA film is 5.67 g cm^−3^, much lower than the reported HEAs. The average hardness is 5.7 GPa and the modulus is 110.7 GPa. The strength and plasticity of the HEA film pillars are 3.6 GPa and 58.0%, respectively, showing an excellent strength–ductility synergy. The high strength is mainly ascribed to the disordered state, the nanostructure, and the lattice distortion effect caused by the different atomic sizes. The large plasticity is affected by the ductile shear band and the island structure. In addition, the AlTiVNb HEA film shows a current density of 4.90 × 10^−8^ A/cm^2^ and a potential of −0.234 V in the 3.5 wt.% NaCl solution, manifesting a better corrosion resistance than that of 316L stainless steels. The superior corrosion resistance is ascribed to the chemical disorder state, cocktail effect, and homogeneous amorphous structure. This finding provides unpreceded opportunities for future applications of flexible microelectronic devices and mechanical metamaterials.

## Data Availability

Not applicable.
